# Understanding local ethnic inequalities in childhood BMI through cross-sectional analysis of routinely collected local data

**DOI:** 10.1186/s12889-019-7870-2

**Published:** 2019-11-28

**Authors:** Marie Murphy, Rebecca Johnson, Nicholas R. Parsons, Wendy Robertson

**Affiliations:** 1 Warwick Medical School, University of Warwick, Coventry, CV4 7AL UK; 2 Coventry University, Coventry, UK

**Keywords:** Childhood, BMI, Weight, Obesity, Ethnicity, Deprivation, Inequalities, Multi-level modelling, School

## Abstract

**Background:**

Local-level analysis of ethnic inequalities in health is lacking, prohibiting a comprehensive understanding of the health needs of local populations and the design of effective health services. Knowledge of ethnic disparities in child weight status is particularly limited by overlooking both the heterogeneity within ethnic groupings; and the complex ecological contexts in which obesity arises. This study aimed to establish whether there was variation in childhood BMI across ethnic groups in Coventry, and the influence of individual, school and neighbourhood contexts, using routinely collected local data.

**Methods:**

National Child Measurement Programme data were compiled for the period 2007/8–2014/15 and combined with routinely collected local data reflecting school performance and demographics, and school and neighbourhood physical environments. Multi-level modelling using Monte Carlo Markov Chain methods was used to account for the clustering of children within schools and neighbourhoods. Ethnic group differences in BMI z-score (zBMI) were explored at 4–5 years and 10–11 years for girls and boys alongside individual, school and neighbourhood covariates.

**Results:**

At age 4–5 years (*n* = 28,407), ethnic group differences were similar for boys and girls, with children from South Asian, White other, Chinese and ‘any other’ ethnic groups having a significantly lower zBMI, and Black African children having a higher zBMI, versus White British (WB) children. Patterns differed considerably at age 10–11 years (*n* = 25,763) with marked sex differences. Boys from White other, Bangladeshi and Black African groups had a significantly higher zBMI than WB boys. For girls, only children from Black ethnic groups showed a significantly higher zBMI. Area-level deprivation was the only important school or neighbourhood covariate, but its inclusion did not explain ethnic group differences in child zBMI.

**Conclusion:**

This analysis contributes to the existing literature by identifying nuanced patterns of ethnic disparities in childhood adiposity in Coventry, supporting the targeting of early obesity prevention for children from Black African groups, as well as girls from Black Caribbean and Black other ethnic backgrounds; and boys from Bangladeshi and White other ethnic backgrounds. It also demonstrates the utility of exploring routinely collected local data sets in building a comprehensive understanding of local population needs.

## Introduction

Childhood obesity prevalence is unequally distributed across ethnic groups in the UK, with cross-sectional and longitudinal analyses finding a pattern of high risk across Black ethnic groups, and a possible increased risk in South Asian children [1–5]. Potential mechanisms underpinning ethnic disparities in childhood obesity are broad, ranging from biological and anthropometric explanations, to environmental, socio-economic and structural barriers and facilitators [6]. Despite a general acknowledgement that these determinants act and interact across multiple levels of influence ranging from the individual child to public policy [7–10], studies have tended to explore the effect of determinants upon weight status at the individual level only.

A small number of studies have begun to explore and account for aggregate variability at the school and neighbourhood level using multi-level analysis [11, 12], however, these studies did not seek to combine a wide range of routinely collected local data to specifically explore the potential differential effects of these two ecological contexts across ethnic groups. In their 2018 resource on tackling ethnic inequalities in health, Public Health England (PHE) highlighted the necessity of understanding local health needs for the effective targeting of policies and services [13].

This study aimed to demonstrate how routinely collected local data can be used to build an understanding of ethnic inequalities in childhood BMI, exploring the role of individual, school and neighbourhood factors upon ethnic group patterns through a multi-level modelling approach.

## Methods

### Setting

Coventry is a city located in the West Midlands of England (52.4068° N, 1.5197° W). With 360,100 residents [14], it is the ninth largest city in England [15] and, as an urban area, is characterised by a multi-ethnic population and by high levels of deprivation. 33.4% of residents are from minority ethnic groups [14], due to historical and recent migration to the city and high birth rates in non- UK-born mothers [16]. Post-war industrial expansion resulted in the migration of South Asian, Irish and Black Caribbean communities to the city. In more recent years, migration to Coventry has originated from Afghanistan, Iraq and Africa and the new accession states in the European Union [16]. The childhood population of Coventry is especially diverse, with 49% of primary school children from minority ethnic backgrounds [17]. In the childhood population, Asian / Asian British is the largest minority ethnic group, with 19% of children from Bangladeshi, Indian, Pakistani or other Asian ethnic backgrounds. There are also many children from Black / Black British, White other and mixed backgrounds. 33% of Coventry primary school children speak a language other than English as their first language [17].

Deprivation is concentrated within the city, with 31% of neighbourhoods (lower super output areas) ranked in the 20% most deprived in the country, mainly in the city centre, north and east of the city [18]. 21% of children in Coventry live in low-income households [14]. The city is typical of many others in England, and more generally in the developed world. Thus we expected the methodology outlined here and the results to have wider application in similar settings elsewhere.

### Data set

All data used in this analysis were made available by the Local Authority, with use permitted through a data processing agreement and honorary contract of the first author with Coventry City Council.

#### National Child Measurement Programme

The National Child Measurement Programme (NCMP) is a national health surveillance programme which measures the heights and weights of children in reception year (aged 4–5 years) and year six (aged 10–11 years) in participating state schools in England. Measurements are taken and recorded by trained staff using a published protocol [19, 20]. Parental consent for child participation in the NCMP is gained through an opt-out process.

Annual data collected from the NCMP for Coventry over the period 2007/8–2014/15 were combined for each year group (reception and year six). NCMP participation in Coventry is typically high, with 98% of eligible children taking part in 2014/15 [21], although this varied slightly across the data period.

Participants were excluded if they had missing or invalid data for ethnicity, index of multiple deprivation (IMD) or lower super output area (LSOA). Those with ethnicity ‘not stated’ and those attending independent schools were also removed. Analysis was restricted to those attending school and residing within the boundaries of Coventry.

#### Individual-level variables

BMI z-score (zBMI) was used as the outcome variable. zBMI describes the standard deviation score of BMI-for-age-and-sex in relation to an external UK90 reference population [22–24]. A zBMI of zero is equivalent to the mean for the UK90 reference population (i.e. indicating a BMI at the 50th centile). Where provided, overweight and obesity was defined using population monitoring cut-offs of ≥85th centile (zBMI = 1.04) for overweight and ≥ 95th centile (zBMI = 1.64) for obese.

Ethnicity forms part of the NCMP data collection procedure and was compiled from the school information management system or child health record based on parental report. Categories were derived from National Health Service classification [25] and were grouped into 12 codes for the purpose of this analysis: White British, White other, mixed ethnicity, Indian, Pakistani, Bangladeshi, any other Asian background, Black Caribbean, Black African, any other Black background, Chinese, and any other ethnic background. Additional individual level covariates included, and collected as part of the NCMP, were sex, age (in months) and year of measurement.

#### School and neighbourhood level variables

School-level variables were selected to reflect aspects of school performance e.g. academic attainment; the physical environment around the school; and pupil characteristics. IMD decile for school postcode is collected as part of the NCMP and was included as a school-level covariate. Pupil intake (number on school roll), proportion of children from Black and minority ethnic groups (% BME), proportion of children with English as a second language (% ESL), and proportion of children achieving level 4 or above in Key Stage 2 tests (% KS2) for each school was obtained from the school census, carried out annually by the Local Authority, for each year of measurement. Ofsted grades were obtained from the Ofsted website, with overall effectiveness ratings used to allocate schools into two categories (good or above; satisfactory or below). Where missing, data from the previous or following year was used as appropriate. The FSA Food Hygiene Rating Scheme list, compiled by the Local Authority, was accessed to identify and map the number of takeaways within a 400 m buffer (straight line radius) surrounding each school using MapInfo Stratus. These data were coded based on the child’s school and appended to the NCMP data set in Stata v14. IMD decile for each child’s postcode is collected as part of the NCMP and was included as a neighbourhood-level covariate.

### Ethical approval

Ethical approval was granted from the University of Warwick Biomedical and Scientific Research Ethics Committee (REGO-2015-1368).

#### Analysis

Data were analysed as a two-level cross-classified multi-level (mixed-effects) linear regression model, with the child as the level one unit and school and home neighbourhood (LSOA) as the level two units. Markov Chain Monte Carlo (MCMC) methods were utilised for parameter estimation as they provide greater flexibility for complex non-hierarchical structures compared to conventional maximum likelihood-based methods [26]. Analyses were stratified by year group and sex and were conducted in Stata v14 using MLWiN v2.36 [27] for multi-level analysis through the runmlwin code [28].

A number of models were created to explore the influence of covariates at each level: 1) a null model with random effects for school and neighbourhood (null model); 2) a model with ethnic group added, to establish the unadjusted zBMI and amount of variance attributable to ethnic group (model 1); 3) a model with retained child, school and neighbourhood level covariates (model 2). Interaction terms for ethnicity with retained covariates were also tested. Covariates were added in a step-wise manner and retained only when model fit was improved, based on a reduction in the Bayesian Deviance Information Criterion (DIC) of greater than five [29]. Covariates that did not improve model fit were excluded in order to provide the best fitting model. Interaction terms were tested in the same way. The proportion of variation explained by the addition of covariates at each level was calculated from the residual error variances for the null model versus the final model. Regression coefficients and variance partition coefficients (VPC) (i.e. the proportion of the total variance accounted for) with 95% credible intervals and *P* values (based on the posterior distributions), are presented in the results, alongside the DIC.

## Results

### Sample description

After removal of observations meeting the exclusion criteria (*n* = 3266), the total sample consisted of 54,170 unique observations (28,407 in reception year and 25,763 in year six) in 84 schools and 197 neighbourhoods. Table 1 displays the sample size, mean zBMI with standard deviation (SD) and percentage overweight or obese with 95% confidence intervals (CI) for individual-level variables and Table 2 displays these for school and neighbourhood-level variables.

**Table 1:** Sample size, mean zBMI and percentage overweight or obese for individual level variables

	Reception	Year 6		Reception		Year 6	
	Sample size (proportion of sample)			zBMI	Overweight/obese (>85th centile)	zBMI	Overweight/obese (>85th centile)
*INDIVIDUAL LEVEL VARIABLES*							
*Sex*	%	%	*Sex*	Mean (SD)	% (95% CI)	Mean (SD)	% (95% CI)
F	49	49	F	0.33 (1.05)	22 (22,23)	0.45 (1.24)	33 (33,34)
M	51	51	M	0.35 (1.13)	24 (24,25)	0.59 (1.23)	37 (36,37)
*Ethnicity*	%	%	*Ethnicity*	Mean (SD)	% (95% CI)	Mean (SD)	% (95% CI)
White British	57	62	White British	0.41 (0.97)	24 (23,24)	0.51 (1.19)	33 (33,34)
White other	6	5	White other	0.28 (1.07)	21 (19,23)	0.57 (1.24)	37 (34,40)
Mixed ethnicity	6	5	Mixed ethnicity	0.33 (1.11)	23 (21,25)	0.58 (1.26)	37 (34,39)
Indian	8	9	Indian	−0.09 (1.32)	17 (16,19)	0.37 (1.41)	35 (33,37)
Pakistani	5	5	Pakistani	0.05 (1.29)	20 (18,22)	0.48 (1.44)	39 (37,42)
Bangladeshi	2	2	Bangladeshi	0.24 (1.35)	27 (24,32)	0.73 (1.37)	46 (41,50)
Other Asian backgrounds	4	3	Other Asian backgrounds	0.17 (1.15)	19 (17,22)	0.50 (1.26)	35 (32,39)
Black Caribbean	1	1	Black Caribbean	0.47 (1.14)	32 (26,38)	0.74 (1.20)	41 (35,47)
Black African	8	6	Black African	0.58 (1.18)	32 (30,34)	0.71 (1.21)	42 (40,45)
Other Black backgrounds	1	1	Other black backgrounds	0.49 (1.22)	32 (27,38)	0.88 (1.15)	44 (36,52)
Chinese	1	0.3	Chinese	−0.03 (0.99)	15 (10,22)	0.16 (1.21)	23 (14,34)
Any other ethnic group	1	1	Any other ethnic group	0.37 (1.25)	25 (21,30)	0.38 (1.23)	30 (24,36)
*Year of measurement*	%	%	*Year of measurement*	Mean (SD)	% (95% CI)	Mean (SD)	% (95% CI)
2007/08	10	12	2007/08	0.38 (1.08)	25 (23,26)	0.47 (1.22)	34 (32,35)
2008/09	11	12	2008/09	0.37 (1.05)	23 (22,25)	0.53 (1.20)	35 (33,36)
2009/10	12	13	2009/10	0.38 (1.06)	23 (22,24)	0.54 (1.23)	35 (34,37)
2010/11	12	13	2010/11	0.38 (1.08)	24 (23,26)	0.56 (1.21)	36 (34,37)
2011/12	13	13	2011/12	0.37 (1.09)	24 (23,26)	0.52 (1.25)	36 (34,37)
2012/13	14	13	2012/13	0.25 (1.07)	21 (19,22)	0.50 (1.26)	34 (33,36)
2013/14	14	12	2013/14	0.29 (1.18)	25 (24,26)	0.53 (1.26)	35 (34,37)
2014/15	15	13	2014/15	0.32 (1.08)	23 (22,24)	0.52 (1.27)	35 (34,37)
*Age*	Mean (95% CI)	Mean (95% CI)	*Age (quantiles)*	Mean (SD)	% (95% CI)	Mean (SD)	% (95% CI)
Age (months)	59.7 (59.7, 59.8)	132 (132.0, 132.1)	1	0.37 (1.08)	24 (23,25)	0.54 (1.22)	35 (34,36)
			2	0.34 (1.09)	23 (22,24)	0.53 (1.22)	35 (33,36)
			3	0.33 (1.08)	23 (22,24)	0.52 (1.26)	35 (34,36)
			4	0.31 (1.11)	23 (22,24)	0.49 (1.25)	35 (34,36)
Total	28,407	25,763	Total	0.34 (1.09)	23 (23,24)	0.52 (1.24)	35 (34,36)

**Table 2:** Sample size, mean zBMI and percentage overweight or obese for school and neighbourhood-level variables

	Reception	Year 6		Reception		Year 6	
	Sample size (proportion of sample)			zBMI	Overweight/obese (>85th centile)	zBMI	Overweight/obese (>85th centile)
*SCHOOL LEVEL VARIABLES*							
*School IMD Quintile*	%	%	*School IMD Quintile*	Mean (SD)	% (95% CI)	Mean (SD)	% (95% CI)
1 (lowest deprivation)	8	2167 (8)	1 (lowest deprivation)	0.14 (1.04)	17 (15,19)	0.35 (1.21)	29 (27,31)
2	15	3836 (15)	2	0.28 (1.00)	20 (19,21)	0.41 (1.17)	30 (28,31)
3	26	6813 (26)	3	0.39 (1.05)	25 (24,26)	0.51 (1.22)	34 (33,35)
4	20	5000 (19)	4	0.39 (1.09)	26 (24,27)	0.57 (1.24)	37 (35,38)
5 (highest deprivation)	33	7947 (31)	5 (highest deprivation)	0.34 (1.16)	24 (23,25)	0.6 (1.29)	39 (37,40)
*Ofsted*	%	%	*Ofsted*	Mean (SD)	% (95% CI)	Mean (SD)	% (95% CI)
Good or above	59	14,938 (58)	Good or above	0.31 (1.09)	23 (22, 23)	0.5 (1.23)	34 (34,35)
Satisfactory or below	42	10,825 (42)	Satisfactory or below	0.37 (1.09)	25 (24, 25)	0.6 (1.25)	36 (35, 37)
	Median (IQR)	Median (IQR)	*Number in school (quantiles)*	Mean (SD)	% (95% CI)	Mean (SD)	% (95% CI)
Number in school	396 (226)	398 (236)	1	0.39 (1.06)	25 (24,26)	0.51 (1.22)	34.3 (33.1,35.5)
			2	0.38 (1.06)	24 (23,25)	0.56 (1.22)	35.8 (34.6,36.9)
			3	0.31 (1.11)	23 (22,24)	0.51 (1.25)	34.7 (33.5,35.9)
			4	0.27 (1.13)	22 (21,23)	0.5 (1.27)	35 (33.9,36.2)
	Median (IQR)	Median (IQR)	*% BME (quantiles)*	Mean (SD)	% (95% CI)	Mean (SD)	% (95% CI)
% BME	38 (31)	36 (30)	1	0.34 (1)	22 (21,23)	0.48 (1.18)	33 (32,34)
			2	0.37 (1.06)	24 (23,25)	0.51 (1.21)	34 (33,35)
			3	0.36 (1.07)	24 (23,25)	0.53 (1.27)	36 (34,37)
			4	0.28 (1.21)	23 (22,24)	0.56 (1.3)	38 (37,39)
	Median (IQR)	Median (IQR)	*% ESL (quantiles)*	Mean (SD)	% (95% CI)	Mean (SD)	% (95% CI)
% ESL	22 (27)	21 (27)	1	0.35 (1)	23 (22,24)	0.47 (1.19)	33 (31,34)
			2	0.38 (1.05)	24 (23,25)	0.51 (1.21)	34 (33,35)
			3	0.35 (1.09)	24 (23,25)	0.55 (1.26)	36 (33,37)
			4	0.28 (1.2)	23 (23,24)	0.55 (1.3)	37 (36,37)
	Median (IQR)	Median (IQR)	*% KS2 (quantiles)*	Mean (SD)	% (95% CI)	Mean (SD)	% (95% CI)
% KS2	75 (18)	75 (19)	1	0.35 (1.11)	24 (23,25)	0.58 (1.27)	37 (36,39)
			2	0.36 (1.09)	24 (23,25)	0.53 (1.25)	36 (33,37)
			3	0.33 (1.1)	23 (22,24)	0.48 (1.22)	33 (32,34)
			4	0.32 (1.06)	23 (22,23)	0.49 (1.2)	33 (32,35)
	Median (IQR)	Median (IQR)	*Takeaways near school (quantiles)*	Mean (SD)	% (95% CI)	Mean (SD)	% (95% CI)
Takeaways near school	2 (4)	2 (4)	1	0.34 (1.07)	23 (22,23)	0.5 (1.23)	34 (33,35)
			2	0.35 (1.1)	24 (23,25)	0.57 (1.26)	37 (35,38)
			3	0.37 (1.06)	24 (23,25)	0.53 (1.21)	35 (34,36)
			4	0.29 (1.16)	23 (22,25)	0.51 (1.28)	35 (34,37)
*NEIGHBOURHOOD LEVEL VARIABLES*							
*Neighbourhood IMD Quintile*	%	%	*Neighbourhood IMD Quintile*	Mean (SD)	% (95% CI)	Mean (SD)	% (95% CI)
1 (lowest deprivation)	4	5	1 (lowest deprivation)	0.04 (1.04)	14.3 (12,17)	0.28 (1.19)	27 (25,29)
2	13	14	2	0.27 (1.01)	20 (19,22)	0.41 (1.18)	31 (29,32)
3	19	20	3	0.34 (1.04)	23 (22,24)	0.49 (1.21)	33 (32,34)
4	24	24	4	0.37 (1.07)	24 (23,25)	0.54 (1.24)	36 (35,37)
5 (highest deprivation)	40	36	5 (highest deprivation)	0.37 (1.14)	25 (24,26)	0.6 (1.28)	38 (37,39)
Total	28,407	25,763	Total	0.34 (1.09)	23 (23,24)	0.52 (1.24)	35 (34,36)

There were significant differences in population characteristics by ethnic group. For example, children from most minority ethnic groups tended to go to schools in the most deprived areas, with the exception of Indian children, Chinese children and those from mixed backgrounds. The schools attended by most Pakistani and Bangladeshi children were located in the most deprived areas (≥67% attended schools in the most deprived quintile, compared to 32% in the whole sample).

Neighbourhood characteristics showed similar patterns for White British, Indian and Chinese children, with a relatively high proportion of these children living in areas of comparatively low deprivation (≤30% in the highest quintile, compared to 38% in the whole sample). The Pakistani, Bangladeshi and Black African groups had the highest proportion of children living in deprived areas (≥69% in the most deprived quintile).

### Multi-level analysis

After the addition of fixed explanatory covariates, the best-fitting mixed-effects regression model differed for reception year and year six, resulting in different covariates being retained in the final models for these 2 year groups.

#### Reception year

**Fig. 1:**
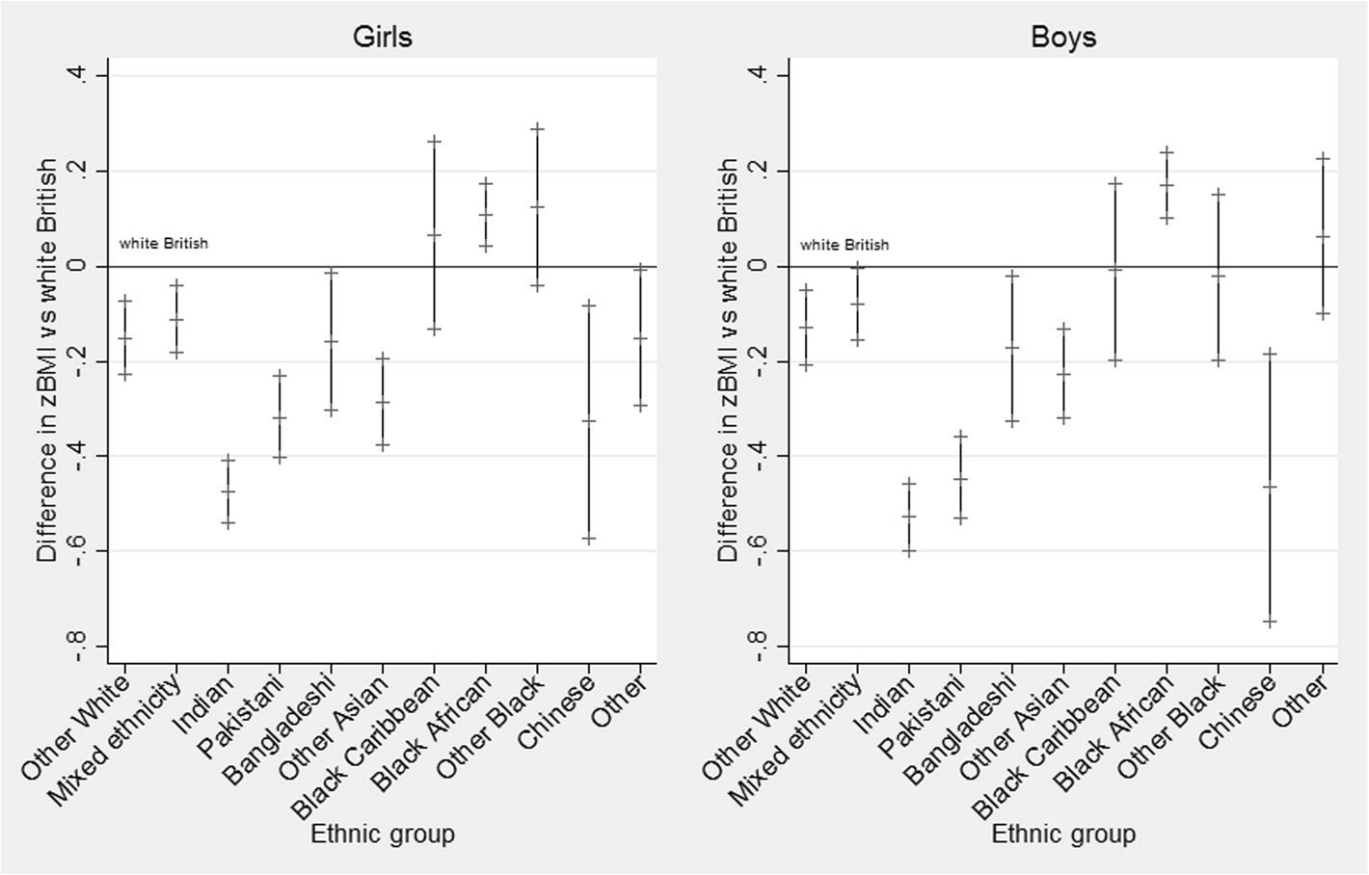
Difference in zBMI for minority ethnic groups versus the White British reference group for reception year girls (left) and boys (right). Zero represents the White British reference group. Regression coefficients for each ethnic group are displayed alongside 95% credible intervals. Results of the fully adjusted model (model 2)

In reception year (aged 4–5 years), ethnic group differences were similar for girls and boys (Fig. 1). Black African children were the only group to have a consistently higher zBMI in this age group. Mean zBMI was higher by 0.11 (95% credible intervals = 0.04, 0.17; *P* = 0.001) for girls and 0.17 (95% credible intervals = 0.1, 0.24; *P* < 0.001) for boys in adjusted models (model 2, Table 3). Children from White other, mixed, Indian, Pakistani, Bangladeshi, other Asian and Chinese groups had a lower zBMI compared to the White British reference group. Boys tended to have a higher zBMI overall compared to girls, with the exception of those from Pakistani, other Black and Chinese ethnic groups (for whom boys had a lower zBMI than girls).

**Table 3:** Regression coefficients and variance partition coefficients for school and neighbourhood for models - reception year

	Girls			Boys		
	Null model	Model 1 ^a^	Model 2 ^b^	Null model	Model 1 ^a^	Model 2 ^b^
*FIXED EFFECTS*	Coefficient [95% credible intervals]	Coefficient [95% credible intervals]	Coefficient [95% credible intervals]	Coefficient [95% credible intervals]	Coefficient [95% credible intervals]	Coefficient [95% credible intervals]
White British (reference)	0.34 [0.31,0.37]	0.41 [0.38,0.45]	0.29 [0.21,0.38]	0.36 [0.32,0.39]	0.43 [0.39,0.46]	0.38 [0.30,0.47]
*Ethnicity*		Coefficients	Coefficients		Coefficients	Coefficients
White other		−0.16 [−0.23,-0.08] *P* < 0.001	−0.15 [−0.23,-0.07] *P* < 0.001		−0.14 [−0.21,-0.06] *P* < 0.001	− 0.13 [− 0.21,-0.05] *P* < 0.001
Mixed ethnicity		−0.11 [− 0.18,-0.03] *P* = 0.002	−0.11 [− 0.18,-0.04] *P* = 0.001		−0.07 [− 0.15,0.00] *P* = 0.034	− 0.08 [− 0.15,-0.00] *P* = 0.019
Indian		− 0.47 [− 0.54,-0.41] *P* < 0.001	− 0.47 [− 0.54,-0.41] *P* < 0.001		− 0.53 [− 0.60,-0.46] *P* < 0.001	−0.53 [− 0.60,-0.46] *P* < 0.001
Pakistani		−0.30 [− 0.39,-0.21] *P* < 0.001	−0.32 [− 0.40,-0.23] *P* < 0.001		− 0.43 [− 0.52,-0.35] *P* < 0.001	−0.45 [− 0.53,-0.36] P < 0.001
Bangladeshi		−0.14 [− 0.28,0.01] *P* = 0.031	−0.16 [− 0.30,-0.01] *P* = 0.016		− 0.15 [− 0.30,0.00] *P* = 0.025	−0.17 [− 0.33,-0.02] *P* = 0.014
Other Asian backgrounds		−0.28 [− 0.38,-0.19] *P* < 0.001	−0.29 [− 0.38,-0.20] P < 0.001		− 0.22 [− 0.32,-0.13] *P* < 0.001	−0.23 [− 0.32,-0.13] *P* < 0.001
Black Caribbean		0.08 [−0.12,0.28] *P* = 0.216	0.07 [− 0.13,0.26] *P* = 0.257		0.01 [− 0.18,0.20] *P* = 0.486	− 0.01 [− 0.20,0.18] *P* = 0.465
Black African		0.12 [0.06,0.19] P < 0.001	0.11 [0.04,0.17] P < 0.001		0.18 [0.11,0.25] *P* < 0.001	0.17 [0.10,0.24] *P* < 0.001
Other Black Backgrounds		0.14 [− 0.03,0.30] *P* = 0.053	0.12 [− 0.04,0.29] *P* = 0.07		−0.02 [− 0.20,0.15] *P* = 0.408	− 0.02 [− 0.20,0.15] *P* = 0.392
Chinese		−0.34 [− 0.60,-0.09] *P* = 0.002	−0.33 [− 0.58,-0.08] *P* = 0.005		−0.48 [− 0.77,-0.20] *P* < 0.001	−0.46 [− 0.75,-0.18] *P* = 0.002
Any other ethnic group		−0.14 [− 0.29,0.01] *P* = 0.033	−0.15 [− 0.30,− 0.01] *P* = 0.022		0.07 [− 0.10,0.23] *P* = 0.214	0.06 [− 0.10,0.22] *P* = 0.231
*Child covariates*			Coefficient [95% credible intervals]			Coefficient [95% credible intervals]
Year of measurement			-0.01 [−0.02,-0.01] *P* = 0.001			−0.02 [− 0.03,-0.01] P < 0.001
*School and neighbourhood covariates*			Coefficient [95% credible intervals]			Coefficient [95% credible intervals]
Neighbourhood IMD decile			0.03 [0.02,0.04] *P* < 0.001			0.02 [0.01,0.03] *P* < 0.001
*RANDOM EFFECTS*						
*Decomposition of variance*	% variance	% variance	% variance	% variance	% variance	% variance
School level VPC	1.4%	1.1%	0.7%	1.4%	0.8%	0.7%
Neighbourhood level VPC	0.6%	0.4%	0.3%	0.4%	0.3%	0.3%
Child level VPC	98.0%	98.5%	99.0%	98.2%	98.9%	99.0%
*MODEL FIT*	DIC	DIC	DIC	DIC	DIC	DIC
Bayesian DIC	40,438	40,179	40,161	44,696	44,379	44,357
**Observations**	13,828	13,828	13,828	14,579	14,579	14,579

^a^Model 1 = Ethnicity only; ^b^Model 2 = ethnicity + year of measurement + neighbourhood IMD

In reception year girls and boys models, year of measurement and neighbourhood IMD were the only covariates that were retained based on their inclusion improving model fit. Year of measurement was negatively correlated with zBMI, indicating a significant decline in zBMI from 2007/8–2014/15 for this age group (model 2, Table 3: a decrease in zBMI of − 0.01 [95% credible intervals = − 0.02,-0.01; *P* = 0.001] for girls and − 0.02 [95% credible intervals = − 0.03,-0.01; *P* < 0.001] for boys per year). Neighbourhood IMD was positively associated with zBMI, after controlling for ethnicity (model 2, Table 3: an increase in zBMI of 0.03 [95% credible intervals = 0.02,0.04; *P* < 0.001] for girls and 0.02 [95% credible intervals = 0.01,0.03; *P* < 0.001] for boys per IMD decile). However, deprivation did not explain ethnic group differences, and the introduction of an interaction term for ethnicity and neighbourhood IMD did not substantially improve model fit.

The school and neighbourhood effects were of a similar magnitude for both girls and boys in reception year, with school accounting for 1.4% of the variation for both groups, and neighbourhood accounting for 0.6 and 0.4% respectively in null models (Table 3). The inclusion of ethnicity, year of measurement and neighbourhood IMD accounted for half of this variance for girls, whilst for boys they accounted for half of the school-level variance and a quarter of the neighbourhood-level variance. Age and school characteristics did not contribute to the predictive power of models so were not retained in the final models.

#### Year six

**Fig. 2:**
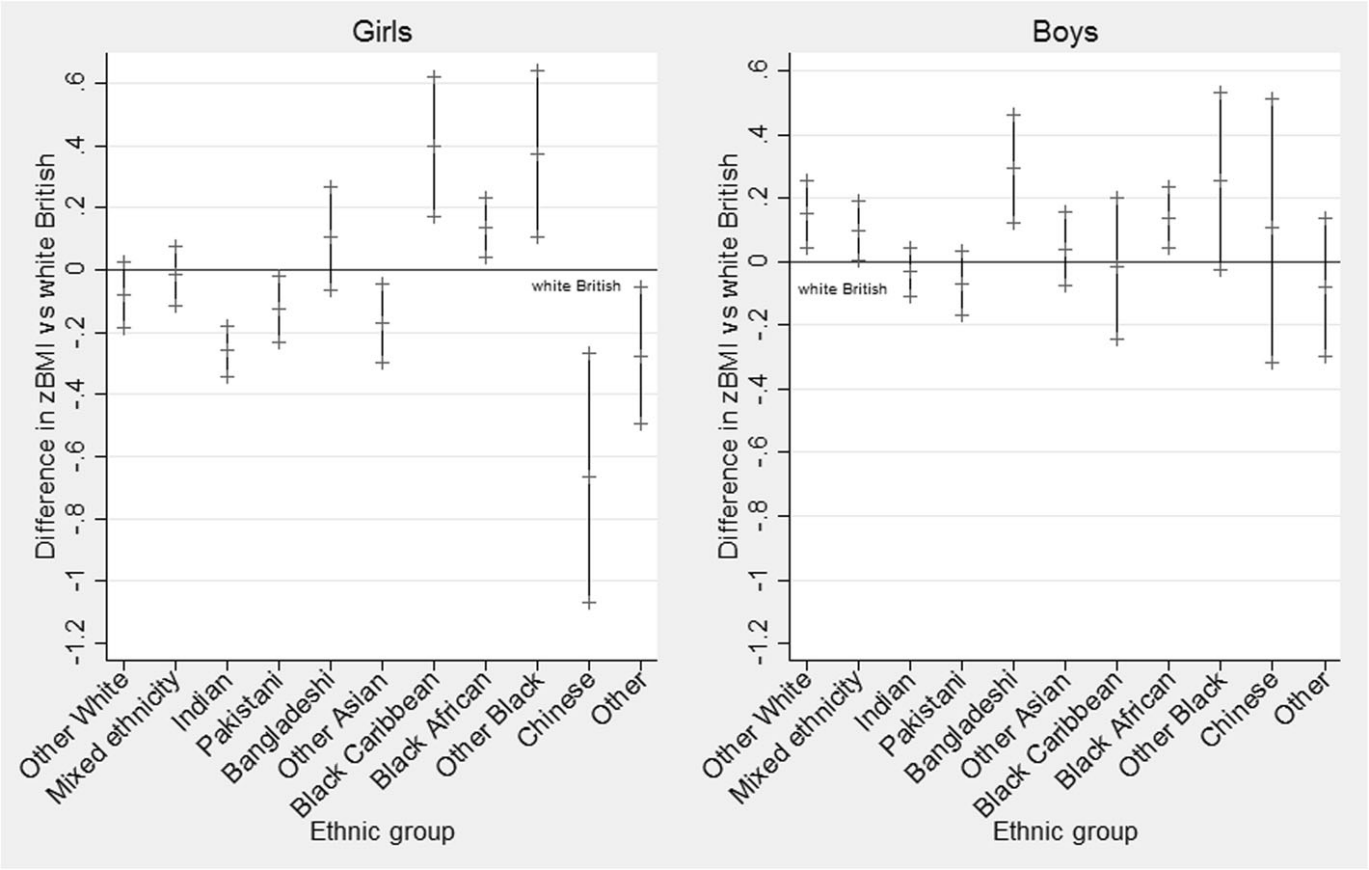
Difference in zBMI for minority ethnic groups versus the White British reference group for year six girls (left) and boys (right). Zero represents the White British reference group. Regression coefficients for each ethnic group are displayed alongside 95% credible intervals. Results of the fully adjusted model (model 2)

As demonstrated in Fig. 2, ethnic differences in zBMI differed considerably by sex in year six (aged 10–11 years). For girls, children from Black ethnic groups (African, Caribbean and other Black backgrounds) had a significantly higher zBMI (by 0.14 [95% credible intervals = 0.04, 0.23; *P* = 0.004]; 0.40 [95% credible intervals = 0.17, 0.62; *P* < 0.001] and 0.37 [95% credible intervals = 0.11, 0.64; *P* = 0.002] respectively for model 2, Table 4). Indian, Pakistani, other Asian and Chinese girls and those from other ethnic backgrounds had a significantly lower zBMI compared to White British girls. Girls from White other and mixed backgrounds did not differ significantly from White British girls. Bangladeshi girls had a significantly higher zBMI versus White British girls in unadjusted models only (0.15 [95% credible intervals = − 0.02, 0.32; *P* = 0.046]), which was fully accounted for by adjustment for deprivation. However, for boys, children from Bangladeshi, Black African, White other and mixed ethnic groups showed a significantly higher zBMI in the adjusted model (by 0.29 [95% credible intervals = 0.12, 0.46; *P* < 0.001]; 0.14 [95% credible intervals = 0.04, 0.24; *P* = 0.001]; 0.15 [95% credible intervals = 0.05, 0.25; P < 0.001]; and 0.10 [95% credible intervals = 0.00, 0.19; *P* = 0.02] respectively for model 2, Table 4). For boys, there were no groups that showed a significantly lower zBMI compared to White British children. As with reception year children, boys in general had a higher zBMI overall compared to girls, with the exception of those from the Black Caribbean group.

**Table 4:** Regression coefficients and variance partition coefficients for school and neighbourhood for models – year six

	Girls			Boys		
	Null model	Model 1 ^a^	Model 2 ^b^	Null model	Model 1 ^a^	Model 2 ^b^
*FIXED EFFECTS*	Coefficient [95% credible intervals]	Coefficient [95% credible intervals]	Coefficient [95% credible intervals]	Coefficient [95% credible intervals]	Coefficient [95% credible intervals]	Coefficient [95% credible intervals]
White British (reference)	0.46 [0.43,0.50]	0.48 [0.44,0.52]	0.18 [0.09,0.27]	0.59 [0.55,0.62]	0.56 [0.52,0.60]	0.39 [0.30,0.49]
*Ethnicity*		Coefficients	Coefficients		Coefficients	Coefficients
White other		−0.05 [−0.15,0.05] *P* = 0.171	−0.08 [−0.18,0.03] *P* = 0.072		0.17 [0.07,0.27] P < 0.001	0.15 [0.05,0.25] P = 0.002
Mixed ethnicity		−0.00 [− 0.10,0.10] P = 0.49	−0.02 [− 0.11,0.08] *P* = 0.368		0.11 [0.02,0.20] P = 0.01	0.10 [0.00,0.19] P = 0.02
Indian		−0.25 [− 0.33,-0.18] P < 0.001	−0.26 [− 0.34,-0.18] *P* < 0.001		−0.03 [− 0.11,0.05] *P* = 0.215	−0.03 [− 0.11,0.04] *P* = 0.205
Pakistani		− 0.08 [− 0.19,0.03] *P* = 0.065	−0.13 [− 0.23,-0.02] *P* = 0.009		−0.04 [− 0.14,0.06] P = 0.214	−0.07 [− 0.17,0.034] *P* = 0.09
Bangladeshi		0.15 [−0.02,0.32] *P* = 0.046	0.10 [− 0.07,0.27] *P* = 0.111		0.33 [0.16,0.50] P < 0.001	0.29 [0.12,0.46] P < 0.001
Other Asian backgrounds		−0.15 [− 0.28,-0.03] *P* = 0.01	−0.17 [− 0.30,-0.05] *P* = 0.004		0.06 [− 0.06,0.17] *P* = 0.168	0.04 [− 0.08,0.16] *P* = 0.251
Black Caribbean		0.41 [0.19,0.64] *P* < 0.001	0.40 [0.17,0.62] *P* < 0.001		0.00 [−0.23,0.23] *P* = 0.496	−0.02 [− 0.24,0.21] *P* = 0.437
Black African		0.19 [0.09,0.29] *P* < 0.001	0.14 [0.04,0.23] P = 0.004		0.17 [0.08,0.27] P < 0.001	0.14 [0.04,0.24] P = 0.003
Other Black Backgrounds		0.41 [0.14,0.68] *P* = 0.001	0.37 [0.11,0.64] *P* = 0.002		0.27 [0.00,0.55] *P* = 0.024	0.25 [−0.02,0.53] *P* = 0.035
Chinese		−0.60 [−1.07,-0.26] *P* = 0.001	− 0.66 [−1.07,-0.26] *P* = 0.001		0.10 [− 0.32,0.52] *P* = 0.317	0.10 [− 0.31,0.52] *P* = 0.312
Any other ethnic group		− 0.24 [− 0.46,0.00] P = 0.022	−0.28 [− 0.50,-0.05] *P* = 0.009		−0.06 [− 0.28,0.16] *P* = 0.302	−0.08 [− 0.30,0.14] *P* = 0.234
*School and neighbourhood covariates*			Coefficient [95% credible intervals]			Coefficient [95% credible intervals]
School IMD decile			0.02 [0.01,0.04] *P* = 0.001			0.01 [−0.01,0.02] *P* = 0.188
Neighbourhood IMD decile			0.02 [0.01,0.04] *P* < 0.001			0.02 [0.01,0.03] *P* = 0.003
*RANDOM EFFECTS*						
*Decomposition of variance*	% variance	% variance	% variance	% variance	% variance	% variance
School level VPC	1.4%	1.2%	0.6%	0.4%	0.5%	0.4%
Neighbourhood level VPC	0.2%	0.2%	0.1%	0.7%	0.7%	0.6%
Child level VPC	98.4%	98.6%	99.3%	98.9%	98.8%	99.0%
*MODEL FIT*	DIC	DIC	DIC	DIC	DIC	DIC
Bayesian DIC	41,352	41,272	41,254	42,615	42,591	42,585
**Observations**	12,667	12,667	12,667	13,096	13,096	13,096

^a^Model 1 = Ethnicity only; ^b^Model 2 =  ethnicity + school IMD + neighbourhood IMD

School and neighbourhood IMD were both retained as the only covariates to improve model fit (Table 4). Neighbourhood deprivation showed a significant relationship with zBMI for girls and boys whilst adjusting for ethnicity (an increase in zBMI by 0.02 [95% credible intervals = 0.01, 0.04; *P* < 0.001] and 0.02 [95% credible intervals = 0.01, 0.03; *P* = 0.003] per IMD decile respectively), whilst school deprivation was significantly correlated with zBMI for girls only (an increase in zBMI by 0.02 [95% credible intervals = 0.01, 0.04; *P* = 0.001] per IMD decile). However, deprivation did not attenuate the effect of ethnicity upon BMI, with two exceptions: firstly, for Pakistani children, who demonstrated a similar zBMI to White British children in unadjusted models, but had a significantly lower zBMI when the model was adjusted for neighbourhood and school IMD (see model 1 versus 2; Table 4); and secondly for Bangladeshi girls, for whom IMD did explain the higher zBMI compared to White British girls (see model 1 versus 2; Table 4). Deprivation also accounted for a substantial amount of the high zBMI in Black African and other Black girls and Bangladeshi boys, but the significantly higher zBMI compared to the White British group remained. The inclusion of interaction terms did not improve model fit.

Although the school and neighbourhood variance remained small, for girls, the school effect was more than three times that of boys (1.4% versus 0.4% respectively in null models). For boys, but not girls, the neighbourhood effect was larger than the school effect (0.7% for boys versus 0.2% for girls). The inclusion of school and neighbourhood IMD did not account for much of the school and neighbourhood variation in zBMI observed in boys (0 and 14% respectively). However, their inclusion did account for half the variation observed across both schools and neighbourhoods for girls. Year of measurement, age, and other school characteristics did not contribute to the predictive power of models so were excluded from the final models.

## Discussion

### Ethnic disparities in child BMI

This study has identified a number of ethnic groups with a significantly higher zBMI compared to the White British reference population, equating to substantial increases in BMI growth chart centile values. For example, controlling for school and neighbourhood IMD, the predicted zBMI for Bangladeshi boys in year six equates to ten centile points higher than the White British reference group (65th centile versus 75th centile). For girls from Black Caribbean and other Black ethnic groups in year six the increased zBMI versus the White British group is the equivalent of 13 and 15 centile points respectively (57th centile versus 70th and 72nd centiles respectively).

Ethnic group disparities in childhood BMI across a 7 year period in Coventry reflect those found in analyses of the national data set from individual years of the NCMP and in a systematic review of the literature [5, 11, 30]. However, this analysis adds to the existing literature on ethnic disparities by identifying more nuanced age- and sex-dependent differences in such patterns. For example, in a systematic review of ethnic inequalities in obesity among British children covering the period 1980–2010, El-Sayed et al. [5] reported an increased risk of obesity in South Asian boys and Black girls and a decreased risk in South Asian girls, relative to ‘Caucasian’ children. The current analysis used disaggregated ethnic groupings to identify an increased zBMI in Black Caribbean and other Black children for year six girls only, and an increased zBMI for year six Bangladeshi boys but not for Pakistani, Indian or other Asian boys, nor those in reception year. In addition, the current study identified an increased zBMI in boys from White other (e.g. White Irish, White Gypsy/Roma and White European) and mixed ethnic groups, which has not been identified elsewhere. Adjusting for deprivation did not explain ethnic differences in zBMI, which suggests that there are additional elements that influence ethnic inequalities in childhood weight status. In a questionnaire study, Falconer et al. [31] found that obesogenic behaviours, including low levels of physical activity, excessive screen time and unhealthy dietary behaviours, were three times more common in Black and South Asian children after adjusting for deprivation, supporting the suggestion that cultural or contextual factors may contribute to these ethnic disparities. For example, low levels of concern for child overweight status in some African groups [32–34] may be partly driven by a cultural valuing of large body size [35, 36] and subsequent lack of recognition of child overweight status [37]. Although migratory background wasn’t directly explored in the current study, the finding that boys from White other ethnic groups have a higher zBMI than those from the White British ethnic group supports the suggestion that migratory background is a potentially influential contextual factor. Many potential mechanisms for the role of migration upon obesity have been proposed [6], however, these have generally focused on migration from low-middle income countries, whereas the majority of White other migrants in Coventry are likely to originate from relatively high income countries e.g. Poland, Ireland.

The sex-related variation in ethnic group patterns of adiposity also demonstrates a potential cultural basis of these findings, for example the increased adiposity observed in White other and Bangladeshi boys but not girls. Although there is little research on dietary behaviours of those from White other ethnic groups, some studies have found less healthy dietary habits in South Asian boys [38]. The current study suggests these behaviours may differ for Bangladeshi boys versus other South Asian groups. The observed variance across Indian, Pakistani, Bangladeshi and other Asian groups supports an aetiology beyond genetic predisposition to metabolic disorder based on shared ancestry. The heterogeneity across South Asian ethnic groups may be related to varying levels of acculturation. Mu’Min Chowdhury et al. [39] found low levels of dietary acculturation in Bangladeshi migrants, typically protective against obesity [40]. However, dietary patterns shifted towards an increase in ‘special menu’ traditional foods following migration, which were typically more energy dense (e.g. biryanis), due to their greater affordability and abundance of ingredients. Kumanyika et al. [41] describe such a scenario as a *cultural-contextual interaction*, in which ‘cultural anchors’ from the past interact with the new context to generate an obesogenic behaviour, in this case, a context where foods previously viewed as treats become abundant and affordable.

Interestingly, neighbourhood deprivation accounted for the high zBMI observed in Bangladeshi girls, but not boys. This differential effect of deprivation may indicate that factors unrelated to socioeconomic status are more influential upon the development of obesity in Bangladeshi boys versus girls. One explanation for gender-based differences may be greater indulgence and permissiveness for boys versus girls in migrating families [42]. Delavari et al. [40] found gender to be a moderating variable in the relationship between dietary acculturation and obesity in adult migrants to high income countries, and the current study suggests this may also be the case in children. However, when considering a potential differential effect of socioeconomic status by gender, it is important to acknowledge that neither the current analysis nor those conducted by Falconer et al. [31] included measures of socioeconomic status at the household and/or individual level. Some residual confounding by socioeconomic status may remain, due to the influence of factors not accounted for in the current analysis.

Metrics of child adiposity may also play a key role in the apparent ethnic disparities in childhood BMI. A key weakness of BMI as a metric is that it is not a direct measure of adiposity. Some studies have found that weight-for-height measures such as BMI underestimate adiposity in South Asian children and overestimate adiposity in Black children [43–47]. Hudda et al. [48] have recently produced a set of adjusted BMI values for children from South Asian and Black African backgrounds, based on direct measures of body fat. Such adjustments may overcome ethnic-specific diagnostic issues in identifying adiposity in UK child populations. BMI also may not fully adjust for the influence of height upon weight in children, so may systematically overestimate the degree of adiposity in tall children [49, 50]. Ethnic group differences in height may therefore account for some of the observed ethnic variation in child BMI and weight status.

The multi-level regression models indicated some degree of clustering at both the school and neighbourhood level, however, the extent to which zBMI varied across schools and especially neighbourhoods was small, with the large majority of variation in zBMI observed at the individual level. The amount of neighbourhood-level variance in the current analysis was similar to that seen in national analyses, yet the amount of variance observed at the school level was substantially lower [11, 12], indicating potential homogeneity across schools in Coventry compared to other areas. Importantly, routinely collected measures of school characteristics and physical environment did not appear influential upon children’s BMI. For example, the current analyses did not find a strong influence of fast food takeaway concentration around schools upon weight status. This may be due to the fact that primary school children have few opportunities to access local shops during or after school, have minimal spending power to purchase from these outlets alone, and experience parental control over eating patterns. For this age group, parental fast food purchases may be more influential upon child dietary behaviours; therefore takeaway density around the child’s home may have a greater influence over zBMI than that around the school [51].

### Strengths and limitations

A strength of this study is that multi-level modelling techniques were utilised to account for the effect of clustering, providing more robust standard errors for the regression coefficients [52]. Seven years of data were combined to increase the sample base and provide more precise coefficient estimates (minimising small number and single-year variation), which allowed exploration across disaggregated ethnic groupings.

This analysis adds to the existing multi-level analyses using NCMP data by exploring ethnic group patterns, and potential interactions between ethnicity and deprivation, as recommended by Townsend et al. [12]; and through the exploration of covariates reflecting school demographics and academic conditions. As encouraged by Dinsdale and Ridler [53], the comparison of local patterns to regional and national ones assists in the targeting of interventions to tackle unhealthy weight among children, and it would be valuable to replicate the methods used here on other regional, as well as the national, NCMP data sets.

A weakness of the study is that the local availability of data limited the covariates that could be included in the analysis. Additional neighbourhood-level data were sought, such as the number of fast food restaurants surrounding the child’s home. However, the transfer of NCMP data sets from the NHS to Local Authorities in 2013 meant that the level of detail required to conduct this analysis (i.e. child postcode) was not consistently accessible to the researchers for the full data period. Additional routine data on school characteristics were also sought, including percentage of children participating in >two hours physical education per week; historical awarding of Healthy Schools status; participation in School Games competitions; and proportion of pupil premium funding spent on physical activity. However, reporting for these data items was either incomplete for the 7 year period or was no longer available for use, prohibiting their inclusion. The incomplete or unavailable nature of these variables was due to the initiation or cessation of surveys or programmes within the 7 year period studied, reflecting the discontinuity of efforts to position obesity-prevention strategies in schools. Overall, despite the potential for a wealth of data to be routinely available at a local level, the practicalities of conducting secondary, retrospective data analysis hampered the building of a more comprehensive model of childhood obesity locally.

The evolution of the NCMP as a surveillance programme was also influential in the way in which the analysis was conducted. For example, until recently it was not possible to track a child’s measurements from reception year to year 6. The inclusion of NHS numbers as unique identifiers now allows tracking of children through primary school, which will provide valuable insights into the relationship between early and late childhood weight status going forward, and will allow cross-referencing with health datasets. Research has been conducted on the value of introducing additional time points for NCMP measurement [54], which may help to build a more complete picture of ethnic inequalities. For example, at what point boys from Bangladeshi backgrounds become at increased risk for obesity.

### Implications and future work

The current findings provide information for the local targeting of obesity prevention and treatment services. This could include for example, the targeting of recruitment for weight management interventions at groups with high risk of adiposity, in particular, boys and girls from Black African backgrounds, older girls from Black Caribbean and other Black ethnic backgrounds, and older boys from Bangladeshi, White other and mixed ethnic backgrounds. This could include additional follow-up from the NCMP, or could be a consideration for school nurses or GPs (General Practitioners) when interacting with families from these ethnic groups. This could also be achieved through tailored recruitment at faith and community centres. In Coventry, families from non-White ethnic groups have historically been over-represented in the Local Authority-delivered weight management service One Body One Life [55], suggesting some appropriate cultural tailoring of recruitment methods and content already occurs. The current analysis provides additional information upon which to target this recruitment. Crucially however, such strategies need to be coupled with an exploration of the unique structural, contextual and cultural factors driving these ethnic group patterns in child weight status locally, particularly through qualitative investigation. In addition, general barriers to GPs and nurses referring families to obesity services need to be explored and addressed [56]. Qualitative approaches would also provide opportunities for parents’ and children’s voices to inform the design of targeted messages or services, creating more “culturally competent” modes of design and delivery and providing detailed understandings of the target communities [57].

This analysis has demonstrated the way in which combinations of routinely collected local data can provide a better understanding of local need in tackling childhood obesity, minimising the need for additional data collection. This approach is in-keeping with one of the four broad approaches to taking local action on health inequalities: knowing your community [13]. Local Authorities may wish to conduct similar analyses in their own regions in order to contribute to local equity audits and needs assessments.

## Conclusions

This analysis contributes to the existing literature by identifying more nuanced patterns of ethnic disparities in childhood adiposity in Coventry, enabled by disaggregated ethnic groupings and stratified analysis by age and sex. The analysis demonstrates the utility of exploring routinely collected local data sets in contributing to a more comprehensive understanding of local population needs. This could be used to better focus obesity prevention services in early childhood at those with the highest need; for example children from Black African groups, girls from Black Caribbean and Black other ethnic backgrounds; and boys from Bangladeshi and White other ethnic backgrounds. However, this should be coupled with qualitative exploration of the contextual and cultural basis of ethnic group patterns, and the acceptability of strategies to tackle childhood obesity with the intended communities. Although in the current study, the supplementary school data added to the NCMP dataset were not retained in final models (e.g. fast food outlet density around schools), the analysis demonstrates ways in which NCMP data could be used to support an understanding of the factors driving childhood overweight and obesity at multiple levels of influence, rather than at the individual-level alone. The methods therefore provide a template for public health analysts in Local Authorities who may wish to replicate the work in their own unique settings. Finally, by using a multi-level modelling approach, this study adds to the growing literature base that acknowledges the school and neighbourhood level aggregate variability in the NCMP dataset.

## Data Availability

The datasets analysed during the current study are not publicly available due to the limits of the data sharing agreement with Coventry City Council, and the risk of indirect identification of individuals.

## Abbreviations

BME: Black and Minority Ethnic Groups
BMI: Body Mass Index
DIC: Deviance Information Criterion
ESL: English as a second language
FSM: Free School Meals
GP: General Practitioner
IMD: Index of Multiple Deprivation
KS2: Key Stage 2
LSOA: Lower super output area
MCMC: Markov Chain Monte Carlo
NIHR: National Institute for Health Research
NCMP: National Child Measurement Programme
PHE: Public Health England
VPC: Variance Partition Coefficient
zBMI: Body Mass Index z-score
